# Supplementary material for: Examination of Signatures of Recent Positive Selection on Genes Involved in Human Sialic Acid Biology
Source: G3 (Bethesda). 2018 Feb 21;8(4):1315–25. doi: 10.1534/g3.118.200035 (PMC5873920; doi:10.1534/g3.118.200035)
Supplement: Supplementary file 1 [file 1315FileS1.docx]

**File S1. Extended Methods**

*1000 Genomes Project Phase 3 Genotype Dataset*

The compressed genotype files (*.vcf.gz) and tab-delimited index (*.tbi) files for all autosomal chromosomes were downloaded from:

<http://ftp.1000genomes.ebi.ac.uk/vol1/ftp/release/20130502/>

We downloaded the human sample panel file that contains information on the ethnicity of each individual included in the genotype dataset from:

<http://ftp.1000genomes.ebi.ac.uk/vol1/ftp/release/20130502/>[integrated_call_samples_v3.20130502.ALL.panel](http://ftp.1000genomes.ebi.ac.uk/vol1/ftp/release/20130502/integrated_call_samples_v3.20130502.ALL.panel)

The 2,504 individuals included in this dataset were divided into five different ethnic groups (i.e., ‘super-populations’) using information in the human sample panel file. We processed the above file to create three ‘population files’ for the three ethnic groups of interest (Africans, Europeans, East Asians), in which each file contains the IDs of individuals belonging to a given ethnic group.

*VCFtools* (v.0.1.13)

*VCFtools* was used to calculate Weir & Cockerham’s *F*_ST_ and to create new VCF files containing genotype data within the protein-coding regions of the sialic acid biology genes. To calculate global *F*_ST_ for each gene, we used the following command:

**./vcftools --gzvcf input.vcf.gz-- chr # --from-bp start_pos --to-bp end_pos --remove-indels --weir-fst-pop AFR_samples_list --weir-fst-pop EUR_samples_list --weir-fst-pop EAS_samples_list --out output**

‘input.vcf.gz’ file refers to the compressed genotype files, sorted by chromosomes, downloaded from the 1000 Genomes Project ftp site. ‘#’ indicates the chromosome id. ‘start_pos’ and ‘end_pos’ refer to the start and end positions of the gene, in base pairs; the coordinates for genes used in the analyses were obtained from Ensembl (GRCh37.p13; release 75) via BioMart and can be found in File S2. The above command will produce two files with the user-defined prefix: *.log file, which stores information on the command used, the sites that were included in the analyses, and the mean and weighted *F*_ST_ values, and *.weir.fst file, which contains *F*_ST_ calculated for each variant, along with the base pair position of that variant. Next, we created new VCF files that contain the genotype data for each gene using the coordinates obtained from Ensembl using the following command:

**./vcf --gzvcf input.vcf.gz --chr # --from-bp start_pos --to-bp end_pos --remove-indels --recode --recode-INFO-all --out output**

This command creates a new VCF (output.recode.vcf) file with genotype data encompassing the regions defined by the ‘--from-bp’ and ‘--to-bp’ arguments. Finally, we created separate VCF files that contain the genotype data for the region that spans 100kb up and downstream of a given sialic acid biology gene for each ethnic group as follows:

**./vcf --gzvcf input.vcf.gz --chr # --from-bp start_pos_100kb --to-bp end_pos_100kb --remove-indels --min-alleles 2 --max-alleles 2 --keep pop_list --recode --recode-INFO-all --out output**

‘start_pos_100kb’ and ‘end_pos_100kb’ refer to positions 100kb upstream and downstream of the start and end positions of the gene as defined by Ensembl, respectively. ‘pop_list’ is the file that lists all the individuals belonging to a given ethnic group. The ‘--min-alleles 2’ and ‘--max-alleles 2’ arguments will filter out multi-allelic sites, as *Selscan* only accepts bi-allelic sites as input data to calculate metrics of extended haplotype homozygosity. As a final step, we also manually filtered out duplicate entries for the same variant in the resulting VCF file. We refer to this file as ‘input_final.vcf’ in the subsequent section (‘*Selscan* (version 1.2.0)’).

*Selscan* (version 1.2.0)

To calculate *nS*_L_ values, we used the following command:

**./selscan --nsl --vcf input_final.vcf --maf 0.01 --threads 16 --out output**

Apart from the minor allele frequency cutoff value (0.01), we used the default settings for other parameters. We used 16 threads to carry out calculations of *nS*_L_ via *Selscan*, as shown above. The above command will create two files with the user-defined prefix: *.nsl.log stores information on specific reason for the variants being excluded from the subsequent calculations, and *.nsl.out is a file that lists values obtained from the *nS*_L_ calculations for each variant, with the 6^th^ column being the unstandardized *nS*_L_ values.

*Tabix* (version.0.2.6)

We used *Tabix* to compress the newly generated VCF files and create tab-delimited index (.tbi) files required for subsequent analyses via *PopGenome*. The commands used are as follows:

**./bgzip -c output.recode.vcf > output.vcf.gz**

**./tabix -p vcf output.vcf.gz**

*SLiM* (version 2.4.1) *and Simulation of Neutral Evolutions*

We used *SLiM* to simulate neutral evolution and generate VCF files of variants resulting from the action of neutral evolution alone, given past demographic events. We used *SLiM*’s implementation of Gravel et al’s pre-computed parameters of human demographic history for our neutral simulations. More specifically, at the beginning of the simulation (i.e. generation 1), the ancestral African effective population size was set to 7,310, which next expanded to 14,474 approximately 148,000 years ago (i.e. 5,920 generations ago). Approximately 51,000 years ago (i.e. 2,040 generations ago), the non-Africans split from Africans; the initial effective population size of these non-Africans was set to 1,861. The migration rates between Africans and non-Africans were set to 15 x 10^-5^. Next, approximately 23,000 years ago (i.e. 920 generations ago), the above-mentioned non-African population split into European and East Asian populations, with the initial effective population size for East Asians set to 554. In the same generation, the European effective population size was reduced to 1,032. The following migration rates were established for the remainder of the simulation: 2.5 x 10^-5^ for between Africans and Europeans, 0.78 x 10^-5^ for between Africans and Asians, and 3.11 x 10^-5^ for between Europeans and East Asians. Between generations 57,080 and 58,000, the European and East Asian populations were set to experience increase in their effective population sizes: for Europeans, the exponential coefficient was 0.0038 and 0.0048 for East Asians. For each gene, we defined the length of the genomic element being simulated as the length of that particular gene. For instance, to simulate *NEU2*, which is 2,386bp long, we used the following argument:

**initializeGenomicElement(g1, 0, 2385)**

After 58,000 generations (i.e. end of the simulation), we sampled 661, 503, and 504 individuals from the “simulated” African, European, and East Asian population, respectively, to match the number of individuals included in the 1000 Genomes Project dataset. We used the following arguments to ensure that pairs of genomes being sampled belonged to the same individual:

**p1_sample = p1.individuals;**

**sampled_p1 = sample(p1_sample, 661);**

**p2_sample = p2.individuals;**

**sampled_p2 = sample(p2_sample, 503);**

**pe_sample = p3.individuals;**

**sampled_p3 = sample(p3_sample, 504);**

p1, p2, and p3, correspond to the simulated African, European, and East Asian populations. Finally, to specify the output format as VCF, we used the arguments as shown below:

**sampled_individuals = c(sampled_p1, sampled_p2, sampled_p3);**

**sampled_individuals.genomes.outputVCF();**

Genomic regions spanning 100kb upstream and downstream of a given gene were created using the same approach as described above. For each gene, we generated 1) 5,000 “neutrally simulated” VCF files that contain genotype information for the “simulated” genic region and 2) an additional 2,500 “neutrally simulated” VCF files that contain genotype information for the “simulated” region that spans 100kb upstream and downstream of a “simulated” sialic acid biology gene. Calculation of Tajima’s *D*, Weir & Cockerham’s *F*_ST_, *nS*_L_, and H12 using these VCF files were carried out as described in previous sections. We refer to these metrics as values generated under “neutral expectations”.
