# Supplementary material for: Examination of Signatures of Recent Positive Selection on Genes Involved in Human Sialic Acid Biology
Source: G3 (Bethesda). 2018 Feb 21;8(4):1315–25. doi: 10.1534/g3.118.200035 (PMC5873920; doi:10.1534/g3.118.200035)
Supplement: Supplementary file 2 [file 1315FileS2.docx]

File S2. Genomic coordinates of the 55 sialic acid biology genes

| **Gene** | **Chr** | **Start (bp)** | **End (bp)** |
| --- | --- | --- | --- |
| *CMAS* | 12 | 22199108 | 22218608 |
| *GNE* | 9 | 36214438 | 36277053 |
| *NAGK* | 2 | 71291474 | 71306935 |
| *NANP* | 20 | 25593571 | 25604811 |
| *NANS* | 9 | 100819021 | 100845357 |
| *NEU1* | 6 | 31825436 | 31830683 |
| *NEU2* | 2 | 233897382 | 233899767 |
| *NEU3* | 11 | 74699179 | 74729938 |
| *NEU4* | 2 | 242749920 | 242758739 |
| *NPL* | 1 | 182758428 | 182799519 |
| *SIAE* | 11 | 124503009 | 124565603 |
| *SLC17A5* | 6 | 74303102 | 74363878 |
| *SLC35A1* | 6 | 88180341 | 88222054 |
| *ST3GAL1* | 8 | 134467091 | 134584183 |
| *ST3GAL2* | 16 | 70413338 | 70473140 |
| *ST3GAL3* | 1 | 44171495 | 44396831 |
| *ST3GAL4* | 11 | 126225535 | 126310239 |
| *ST3GAL5* | 2 | 86066267 | 86116137 |
| *ST3GAL6* | 3 | 98451080 | 98540045 |
| *ST6GAL1* | 3 | 186648274 | 186796341 |
| *ST6GAL2* | 2 | 107418056 | 107503564 |
| *ST6GALNAC1* | 17 | 74620843 | 74639920 |
| *ST6GALNAC2* | 17 | 74559792 | 74583038 |
| *ST6GALNAC3* | 1 | 76540404 | 77100286 |
| *ST6GALNAC4* | 9 | 130670165 | 130679317 |
| *ST6GALNAC5* | 1 | 77333126 | 77531396 |
| *ST6GALNAC6* | 9 | 130647600 | 130667687 |
| *ST8SIA1* | 12 | 22216707 | 22589975 |
| *ST8SIA2* | 15 | 92937058 | 93011958 |
| *ST8SIA3* | 18 | 55018044 | 55038962 |
| *ST8SIA4* | 5 | 100142639 | 100238970 |
| *ST8SIA5* | 18 | 44259081 | 44339220 |
| *ST8SIA6* | 10 | 17360382 | 17496329 |
| *SELE* | 1 | 169691781 | 169733846 |
| *SELL* | 1 | 169659808 | 169680839 |
| *SELP* | 1 | 169558087 | 169599431 |
| *SIGLEC12* | 19 | 51994611 | 52005043 |
| *LAMA1* | 18 | 6941743 | 7117813 |
| *LAMA2* | 6 | 129204342 | 129837714 |
| *CFH* | 1 | 196621008 | 196716634 |
| *CTSA* | 20 | 44518783 | 44527459 |
| *CD22* | 19 | 35810164 | 35838258 |
| *CD33* | 19 | 51728320 | 51747115 |
| *MAG* | 19 | 35783028 | 35804707 |
| *SIGLEC1* | 20 | 3667617 | 3687775 |
| *SIGLEC10* | 19 | 51913275 | 51921057 |
| *SIGLEC11* | 19 | 50452242 | 50464429 |
| *SIGLEC14* | 19 | 52145806 | 52150054 |
| *SIGLEC15* | 18 | 43405477 | 43424045 |
| *SIGLEC16* | 19 | 50472857 | 50479071 |
| *SIGLEC5* | 19 | 52114781 | 52150151 |
| *SIGLEC6* | 19 | 52022779 | 52035110 |
| *SIGLEC7* | 19 | 51645556 | 51656783 |
| *SIGLEC8* | 19 | 51954101 | 51961710 |
| *SIGLEC9* | 19 | 51628165 | 51639908 |

Note. Genomic coordinates were downloaded from Ensembl (GRCh37; release 75) via the BioMart feature.
