# Supplementary material for: Examination of Signatures of Recent Positive Selection on Genes Involved in Human Sialic Acid Biology
Source: G3 (Bethesda). 2018 Feb 21;8(4):1315–25. doi: 10.1534/g3.118.200035 (PMC5873920; doi:10.1534/g3.118.200035)
Supplement: Supplementary file 3 [file 1315FileS3.docx]

**Supplementary Tables**

**Table S1. Values of the four metrics calculated across all 1,668 individuals for the sialic acid biology genes**

| **Gene** | **Tajima’s *D*** | | **Weir & Cockerham’s *F*_ST_** | | | **H12­** | | **Nucleotide Diversity** |
| --- | --- | --- | --- | --- | --- | --- | --- | --- |
|  | ***D*** | ***p*-value** | **Weighted** | **Mean** | ***p*-value** | **H12** | ***p*-value** | **Π** |
| **1) Biosynthesis** | | | | | | | |  |
| ***GNE*** | -1.547 | 0.891 | 0.097 | 0.017 | 0.860 | 0.007 | 0.323 | 0.0009 |
| ***NAGK*** | -1.367 | 0.893 | 0.109 | 0.022 | 0.696 | 0.061 | 0.683 | 0.0012 |
| ***NANP*** | -1.298 | 0.910 | 0.282 | 0.042 | 0.045 | 0.136 | 0.256 | 0.0010 |
| ***­NANS*** | -1.845 | 0.410 | 0.242 | 0.022 | 0.084 | 0.148 | 0.0004 | 0.0007 |
| **2) Activation, Transport, Transfer** | | | | | | | |  |
| ***CMAS*** | -1.613 | 0.720 | 0.177 | 0.024 | 0.255 | 0.04 | 0.760 | 0.0007 |
| ***­SLC35A1*** | -1.644 | 0.741 | 0.039 | 0.008 | 1.000 | 0.048 | 0.001 | 0.0009 |
| ***ST3GAL1*** | -1.479 | 0.976 | 0.120 | 0.022 | 0.715 | 0.0003 | 1.000 | 0.0012 |
| ***ST3GAL2*** | -2.256 | 0.003 | 0.207 | 0.022 | 0.131 | 0.006 | 0.727 | 0.0004 |
| ***ST3GAL3*** | -1.611 | 0.935 | 0.148 | 0.022 | 0.456 | 0.0004 | 1.000 | 0.0008 |
| ***ST3GAL4*** | -1.583 | 0.874 | 0.136 | 0.022 | 0.556 | 0.001 | 1.000 | 0.0010 |
| ***ST3GAL5*** | -1.376 | 0.970 | 0.074 | 0.016 | 0.965 | 0.032 | 0.002 | 0.0010 |
| ***ST3GAL6*** | -1.681 | 0.734 | 0.085 | 0.013 | 0.953 | 0.003 | 0.237 | 0.0008 |
| ***ST6GAL1*** | -1.260 | 1.000 | 0.115 | 0.025 | 0.788 | 0­.0004 | 1.000 | 0.0013 |
| ***ST6GAL2*** | -1.479 | 0.957 | 0.143 | 0.024 | 0.483 | 0.003 | 0.380 | 0.0010 |
| ***ST6GALNAC1*** | -1.437 | 0.872 | 0.116 | 0.021 | 0.661 | 0.112 | 0.032 | 0.0011 |
| ***ST6GALNAC2*** | -1.176 | 0.983 | 0.126 | 0.025 | 0.577 | 0.007 | 1.000 | 0.0016 |
| ***ST6GALNAC3*** | -1.561 | 0.998 | 0.168 | 0.026 | 0.240 | 0.0003 | 1.000 | 0.0009 |
| ***ST6GALNAC4*** | -1.947 | 0.268 | 0.148 | 0.020 | 0.403 | 0.034 | 1.000 | 0.0007 |
| ***ST6GALNAC5*** | -1.734 | 0.669 | 0.110 | 0.019 | 0.858 | 0.001 | 1.000 | 0.0008 |
| ***ST6GALNAC6*** | -2.063 | 0.124 | 0.146 | 0.020 | 0.446 | 0.036 | 0.830 | 0.0005 |
| ***ST8SIA1*** | -1.511 | 0.999 | 0.115 | 0.022 | 0.867 | 0.0003 | 1.000 | 0.0010 |
| ***ST8SIA2*** | -1.325 | 0.994 | 0.138 | 0.025 | 0.531 | 0.001 | 1.000 | 0.0012 |
| ***ST8SIA3*** | -1.605 | 0.741 | 0.156 | 0.027 | 0.368 | 0.036 | 0.778 | 0.0008 |
| ***ST8SIA4*** | -1.824 | 0.414 | 0.077 | 0.014 | 0.975 | 0.004 | 0.015 | 0.0006 |
| ***ST8SIA5*** | -1.395 | 0.983 | 0.089 | 0.020 | 0.918 | 0.001 | 1.000 | 0.0011 |
| ***ST8SIA6*** | -1.261 | 1.000 | 0.139 | 0.025 | 0.531 | 0.001 | 1.000 | 0.0013 |
| **3) Recognition** | | | | | | | |  |
| ***CD22*** | -1.728 | 0.579 | 0.074 | 0.018 | 0.951 | 0.005 | 1.000 | 0.0009 |
| ***CD33*** | -1.913 | 0.313 | 0.124 | 0.019 | 0.593 | 0.053 | 0.531 | 0.0006 |
| ***CFH*** | -1.630 | 0.822 | 0.145 | 0.025 | 0.480 | 0.003 | 0.061 | 0.0009 |
| ***LAMA1*** | -1.378 | 1.000 | 0.187 | 0.032 | 0.166 | 0.0003 | 1.000 | 0.0014 |
| ***LAMA2*** | -1.629 | 0.987 | 0.165 | 0.024 | 0.268 | 0.0003 | 1.000 | 0.0009 |
| ***MAG*** | -1.927 | 0.283 | 0.085 | 0.016 | 0.866 | 0.034 | 0.774 | 0.0007 |
| ***SELE*** | -1.192 | 0.996 | 0.071 | 0.024 | 0.967 | 0.022 | 0.101 | 0.0015 |
| ***SELL*** | -1.335 | 0.933 | 0.081 | 0.019 | 0.895 | 0.034 | 0.829 | 0.0012 |
| ***SELP*** | -1.449 | 0.924 | 0.268 | 0.036 | 0.035 | 0.001 | 1.000 | 0.0010 |
| ***SIGLEC1*** | -1.390 | 0.907 | 0.078 | 0.019 | 0.912 | 0.023 | 0.994 | 0.0012 |
| ***SIGLEC10*** | -1.644 | 0.623 | 0.107 | 0.017 | 0.673 | 0.129 | 0.703 | 0.0011 |
| ***SIGLEC11*** | -1.974 | 0.246 | 0.094 | 0.018 | 0.778 | 0.119 | 0.291 | 0.0007 |
| ***SIGLEC12*** | -0.886 | 0.988 | 0.081 | 0.021 | 0.854 | 0.074 | 0.886 | 0.0022 |
| ***SIGLEC14*** | -0.798 | 0.971 | 0.118 | 0.028 | 0.567 | 0.146 | 0.962 | 0.0015 |
| ***SIGLEC15*** | -1.793 | 0.485 | 0.121 | 0.020 | 0.616 | 0.093 | 0.104 | 0.0008 |
| ***SIGLEC16*** | -0.707 | 0.990 | 0.067 | 0.020 | 0.909 | 0.125 | 0.892 | 0.0027 |
| ***SIGLEC5*** | -1.241 | 0.983 | 0.170 | 0.034 | 0.300 | 0.004 | 1.000 | 0.0012 |
| ***SIGLEC6*** | -1.086 | 0.972 | 0.047 | 0.012 | 0.985 | 0.210 | 0.023 | 0.0015 |
| ***SIGLEC7*** | -2.023 | 0.187 | 0.142 | 0.016 | 0.448 | 0.184 | 0.073 | 0.0005 |
| ***SIGLEC8*** | -1.798 | 0.446 | 0.135 | 0.014 | 0.481 | 0.159 | 0.509 | 0.0009 |
| ***SIGLEC9*** | -1.716 | 0.574 | 0.175 | 0.027 | 0.284 | 0.063 | 0.911 | 0.0010 |
| **4) Recycling, Degradation** | | | | | | | |  |
| ***CTSA*** | -1.712 | 0.560 | 0.064 | 0.012 | 0.934 | 0.183 | 0.248 | 0.0008 |
| ***NEU1*** | -1.999 | 0.196 | 0.086 | 0.009 | 0.779 | 0.494 | 0.027 | 0.0003 |
| ***NEU2*** | -1.948 | 0.181 | 0.128 | 0.012 | 0.469 | 0.493 | 0.475 | 0.0008 |
| ***NEU3*** | -2.043 | 0.122 | 0.116 | 0.014 | 0.679 | 0.098 | 0.001 | 0.0005 |
| ***NEU4*** | -1.081 | 0.956 | 0.099 | 0.023 | 0.731 | 0.017 | 1.000 | 0.0022 |
| ***NPL*** | -1.913 | 0.278 | 0.189 | 0.028 | 0.201 | 0.062 | 0.0004 | 0.0006 |
| ***SIAE*** | -2.136 | 0.024 | 0.225 | 0.024 | 0.085 | 0.017 | 0.001 | 0.0005 |
| ***SLC17A5*** | -0.603 | 1.000 | 0.117 | 0.030 | 0.709 | 0.009 | 0.102 | 0.0019 |

Note. The genes are divided according to the functional categories they belong to. All Tajima’s *D*, weighted and mean *F*_ST_, H12, and *p*-values were rounded to the nearest third decimal place. All π values were rounded to the nearest fourth decimal place. The *p*-values for Tajima’s *D*, weighted *F*_ST_, and H12 values were defined as the proportion of ‘neutrally expected’ values that were more extreme than the observed values.

**Table S2. Values of *nS*_L_ calculated in the three ethnic groups for the sialic acid biology genes**

| **Gene** | **Africans** | | **Europeans** | | **East Asians** | |
| --- | --- | --- | --- | --- | --- | --- |
|  | **Max *nS*_L_** | ***p*-value** | **Max *nS*_L_** | ***p*-value** | **Max *nS*_L_** | ***p*-value** |
| **1) Biosynthesis** | | | | | | |
| ***GNE*** | 1.648 | 0.667 | 1.155 | 0.846 | 1.274 | 0.295 |
| ***NAGK*** | 1.696 | 0.342 | 1.245 | 0.573 | 1.280 | 0.242 |
| ***NANP*** | 1.673 | 0.443 | 1.128 | 0.854 | 0.911 | 0.971 |
| ***NANS*** | 1.437 | 0.999 | 1.387 | 0.174 | 1.290 | 0.214 |
| **2) Activation, Transport, Transfer** | | | | | | |
| ***CMAS*** | 1.791 | 0.074 | 1.454 | 0.057 | 1.273 | 0.257 |
| ***SLC35A1*** | 1.469 | 0.997 | 1.121 | 0.878 | 1.102 | 0.769 |
| ***ST3GAL1*** | 1.733 | 0.293 | 1.398 | 0.185 | 1.361 | 0.139 |
| ***ST3GAL2*** | 1.341 | 1.000 | 0.769 | 1.000 | 0.740 | 0.999 |
| ***ST3GAL3*** | 1.351 | 1.000 | 1.108 | 0.966 | 1.089 | 0.917 |
| ***ST3GAL4*** | 1.558 | 0.951 | 1.399 | 0.164 | 1.197 | 0.568 |
| ***ST3GAL5*** | 1.575 | 0.894 | 1.211 | 0.704 | 1.166 | 0.602 |
| ***ST3GAL6*** | 1.581 | 0.910 | 1.183 | 0.806 | 0.935 | 0.976 |
| ***ST6GAL1*** | 1.670 | 0.642 | 1.460 | 0.081 | 1.595 | 0.001 |
| ***ST6GAL2*** | 1.514 | 0.987 | 1.026 | 0.981 | 1.013 | 0.927 |
| ***ST6GALNAC1*** | 1.957 | 0.002 | 1.178 | 0.749 | 1.290 | 0.228 |
| ***ST6GALNAC2*** | 1.957 | 0.001 | 1.178 | 0.748 | 1.225 | 0.381 |
| ***ST6GALNAC3*** | 1.634 | 0.968 | 1.254 | 0.896 | 1.147 | 0.922 |
| ***ST6GALNAC4*** | 1.379 | 0.999 | 0.928 | 0.992 | 1.250 | 0.319 |
| ***ST6GALNAC5*** | 1.652 | 0.786 | 1.336 | 0.439 | 1.454 | 0.030 |
| ***ST6GALNAC6*** | 1.379 | 1.000 | 0.947 | 0.987 | 1.250 | 0.332 |
| ***ST8SIA1*** | 1.791 | 0.172 | 1.454 | 0.141 | 1.273 | 0.505 |
| ***ST8SIA2*** | 1.683 | 0.473 | 1.309 | 0.417 | 1.217 | 0.479 |
| ***ST8SIA3*** | 1.631 | 0.660 | 1.112 | 0.887 | 0.917 | 0.972 |
| ***ST8SIA4*** | 1.424 | 0.999 | 0.911 | 0.999 | 0.868 | 0.993 |
| ***ST8SIA5*** | 1.440 | 0.999 | 1.183 | 0.804 | 1.174 | 0.605 |
| ***ST8SIA6*** | 1.569 | 0.959 | 1.210 | 0.802 | 1.254 | 0.429 |
| **3) Recognition** | | | | | | |
| ***CD22*** | 1.457 | 0.995 | 1.438 | 0.077 | 1.402 | 0.046 |
| ***CD33*** | 1.811 | 0.052 | 1.115 | 0.894 | 0.969 | 0.932 |
| ***CFH*** | 1.728 | 0.304 | 1.403 | 0.168 | 1.096 | 0.818 |
| ***LAMA1*** | 1.732 | 0.341 | 1.221 | 0.819 | 1.234 | 0.527 |
| ***LAMA2*** | 1.896 | 0.018 | 1.177 | 0.986 | 0.989 | 0.998 |
| ***MAG*** | 1.457 | 0.995 | 1.438 | 0.077 | 1.402 | 0.046 |
| ***SIGLEC1*** | 1.726 | 0.249 | 1.276 | 0.471 | 1.263 | 0.296 |
| ***SIGLEC10*** | 1.939 | 0.002 | 1.161 | 0.778 | 1.239 | 0.356 |
| ***SIGLEC11*** | 1.714 | 0.257 | 1.154 | 0.806 | 1.177 | 0.531 |
| ***SIGLEC12*** | 1.672 | 0.447 | 2.129 | 0.0004 | 1.217 | 0.394 |
| ***SIGLEC14*** | 1.672 | 0.447 | 2.129 | 0.0004 | 1.371 | 0.069 |
| ***SIGLEC15*** | 1.660 | 0.507 | 0.997 | 0.971 | 0.931 | 0.958 |
| ***SIGLEC16*** | 1.714 | 0.253 | 1.154 | 0.798 | 1.177 | 0.532 |
| ***SIGLEC5*** | 1.672 | 0.489 | 2.129 | 0.0004 | 1.371 | 0.079 |
| ***SIGLEC6*** | 1.672 | 0.446 | 2.129 | 0.0004 | 1.217 | 0.390 |
| ***SIGLEC7*** | 1.699 | 0.337 | 1.267 | 0.504 | 1.131 | 0.660 |
| ***SIGLEC8*** | 1.939 | 0.002 | 1.161 | 0.784 | 1.217 | 0.422 |
| ***SIGLEC9*** | 1.699 | 0.337 | 1.267 | 0.485 | 1.131 | 0.658 |
| **4) Recycling, Degradation** | | | | | | |
| ***CTSA*** | 1.576 | 0.838 | 1.049 | 0.939 | 1.301 | 0.183 |
| ***NEU1*** | 1.419 | 0.997 | 1.270 | 0.479 | 1.263 | 0.258 |
| ***NEU2*** | 1.722 | 0.224 | 1.474 | 0.036 | 1.297 | 0.190 |
| ***NEU3*** | 1.356 | 1.000 | 1.054 | 0.948 | 0.965 | 0.952 |
| ***NEU4*** | 1.713 | 0.273 | 1.221 | 0.620 | 1.348 | 0.106 |
| ***NPL*** | 1.362 | 1.000 | 1.129 | 0.880 | 0.905 | 0.978 |
| ***SIAE*** | 1.672 | 0.544 | 1.159 | 0.846 | 1.220 | 0.459 |
| ***SLC17A5*** | 1.509 | 0.989 | 1.096 | 0.926 | 1.164 | 0.641 |

Note. The genes are divided according to the functional categories they belong to. For each gene, *nS*_L_ values were calculated across the region expanding 100kb upstream and downstream. We present un-standardized values. For each gene, the maximum absolute *nS*_L_ value of the entire window is shown here. All *nS*_L_ values and *p*-values were rounded to the nearest third decimal place. The *p*-values in each population were defined as the proportion of ‘neutrally expected’ values that were more extreme than the observed values.

**Table S3.** **Comparisons of the calculated metrics across functional categories of sialic acid biology genes**

**A) Nucleotide Diversity**

| **Pairwise Comparisons** | *U* value | | Adjusted *p*-value |
| --- | --- | --- | --- |
| **Biosynthesis vs Activation, Transport, Transfer** | | 43 | 1.000 |
| **Biosynthesis vs Recognition** | | 32 | 1.000 |
| **Biosynthesis vs Recycling, Degradation** | | 22 | 1.000 |
| **Activation, Transport, Transfer vs Recognition** | | 179 | 1.000 |
| **Activation, Transport, Transfer vs Recycling, Degradation** | | 114 | 1.000 |
| **Recognition vs Recycling, Degradation** | | 119 | 0.556 |

**B) Tajima’s *D***

| **Pairwise Comparisons** | *U* value | | Adjusted *p*-value |
| --- | --- | --- | --- |
| **Biosynthesis vs Activation, Transport, Transfer** | | 52 | 1.000 |
| **Biosynthesis vs Recognition** | | 44 | 1.000 |
| **Biosynthesis vs Recycling, Degradation** | | 23 | 1.000 |
| **Activation, Transport, Transfer vs Recognition** | | 217 | 1.000 |
| **Activation, Transport, Transfer vs Recycling, Degradation** | | 117 | 1.000 |
| **Recognition vs Recycling, Degradation** | | 114 | 0.917 |

**C) Weir & Cockerham’s *F*_ST_**

| **Pairwise Comparisons** | *U* value | | Adjusted *p*-value |
| --- | --- | --- | --- |
| **Biosynthesis vs Activation, Transport, Transfer** | | 54 | 1.000 |
| **Biosynthesis vs Recognition** | | 60 | 1.000 |
| **Biosynthesis vs Recycling, Degradation** | | 21 | 1.000 |
| **Activation, Transport, Transfer vs Recognition** | | 263 | 1.000 |
| **Activation, Transport, Transfer vs Recycling, Degradation** | | 94 | 1.000 |
| **Recognition vs Recycling, Degradation** | | 77 | 1.000 |

**D) *nS*_L_ (Africans)**

| **Pairwise Comparisons** | *U* value | | Adjusted *p*-value |
| --- | --- | --- | --- |
| **Biosynthesis vs Activation, Transport, Transfer** | | 52 | 1.000 |
| **Biosynthesis vs Recognition** | | 23 | 1.000 |
| **Biosynthesis vs Recycling, Degradation** | | 20 | 1.000 |
| **Activation, Transport, Transfer vs Recognition** | | 127 | 0.064 |
| **Activation, Transport, Transfer vs Recycling, Degradation** | | 100 | 1.000 |
| **Recognition vs Recycling, Degradation** | | 133 | 0.089 |

**(E) *nS*_L_ (Europeans)**

| **Pairwise Comparisons** | *U* value | | Adjusted *p*-value |
| --- | --- | --- | --- |
| **Biosynthesis vs Activation, Transport, Transfer** | | 47 | 1.000 |
| **Biosynthesis vs Recognition** | | 28 | 1.000 |
| **Biosynthesis vs Recycling, Degradation** | | 20 | 1.000 |
| **Activation, Transport, Transfer vs Recognition** | | 162 | 0.569 |
| **Activation, Transport, Transfer vs Recycling, Degradation** | | 94 | 1.000 |
| **Recognition vs Recycling, Degradation** | | 124 | 0.304 |

**(F) *nS*_L_ (East Asians)**

| **Pairwise Comparisons** | *U* value | | Adjusted *p*-value |
| --- | --- | --- | --- |
| **Biosynthesis vs Activation, Transport, Transfer** | | 57 | 1.000 |
| **Biosynthesis vs Recognition** | | 45 | 1.000 |
| **Biosynthesis vs Recycling, Degradation** | | 16 | 1.000 |
| **Activation, Transport, Transfer vs Recognition** | | 203 | 1.000 |
| **Activation, Transport, Transfer vs Recycling, Degradation** | | 77 | 1.000 |
| **Recognition vs Recycling, Degradation** | | 91 | 1.000 |

**(G) H12**

| **Pairwise Comparisons** | *U* value | | Adjusted *p*-value |
| --- | --- | --- | --- |
| **Biosynthesis vs Activation, Transport, Transfer** | | 79 | 0.057 |
| **Biosynthesis vs Recognition** | | 52 | 1.000 |
| **Biosynthesis vs Recycling, Degradation** | | 13 | 1.000 |
| **Activation, Transport, Transfer vs Recognition** | | 114 | **0.023*** |
| **Activation, Transport, Transfer vs Recycling, Degradation** | | 23 | **0.008*** |
| **Recognition vs Recycling, Degradation** | | 64 | 1.000 |

Note. For all metrics, pairwise comparisons between functional categories were conducted using two-tailed Mann-Whitney *U* tests. *P*-values were adjusted *post hoc* via the Bonferroni method. Statistically significant comparisons are shown in bold and indicated with an asterisk (*).
